# Facile Synthesis of Hierarchical CoSeO_3_‧2H_2_O Nanoflowers Assembled by Nanosheets as a Novel Anode Material for High-Performance Lithium-Ion Batteries

**DOI:** 10.3390/nano12142474

**Published:** 2022-07-19

**Authors:** Xiao-Xu Ji, Qing-Huai Zhao, Hao Chen, Xin-Wei Luo, Yi Shang, Xiao-Di Liu

**Affiliations:** 1 College of Physics and Electronic Engineering, Nanyang Normal University, Nanyang 473061, China; xxji2010@163.com (X.-X.J.); zqh1262022@126.com (Q.-H.Z.); 2 College of Chemistry and Pharmaceutical Engineering, Nanyang Normal University, Nanyang 473061, China; xiaohao819@126.com (H.C.); 17527755065@163.com (X.-W.L.); s3030199101@163.com (Y.S.)

**Keywords:** hydrothermal method, CoSeO_3_‧2H_2_O, nanoflowers, nanosheets, lithium-ion batteries

## Abstract

As novel anodic materials for lithium-ion batteries (LIBs), transitional metal selenites can transform into metal oxide/selenide heterostructures in the first cycle, which helps to enhance the Li^+^ storage performance, especially in terms of high discharge capacity. Herein, well-defined hierarchical CoSeO_3_‧2H_2_O nanoflowers assembled using 10 nm-thick nanosheets are successfully synthesized via a facile one-step hydrothermal method. When used as anodic materials for LIBs, the CoSeO_3_‧2H_2_O nanoflowers exhibit a considerably high discharge capacity of 1064.1 mAh g^−1^ at a current density of 0.1 A g^−1^. In addition, the obtained anode possesses good rate capability and cycling stability. Owing to the superior electrochemical properties, the CoSeO_3_‧2H_2_O nanoflowers would serve as promising anodic materials for high-performance LIBs.

## 1. Introduction

With the increasing demands of portable electronics and electric vehicles, lithium-ion batteries (LIBs) with high power density and good cycle stability urgently need to be developed [1,2,3,4]. The commercial graphite anodic material is limited to acquiring the requirement of high power density owing to its low theoretical capacity (372 mAh g^−1^) [5,6]. Thus, much effort has been made to explore novel and effective anodes for high-performance LIBs.

In the last few years, transitional metal selenides have received considerable attention for their rich redox active sites, high electronic conductivity, and large theoretical capacities, thus leading to superior electrochemical performance. Accordingly, transitional metal selenides are promising anodes of LIBs [7,8,9,10]. On the other hand, heterostructures composed of materials with different bandgaps can form an internal electric field at the heterointerface, resulting in facilitated charge transport and enhanced surface reaction kinetics [11,12,13,14,15]. For instance, SnS/SnO_2_ can form an electric field in the nanocrystal, so they possess much lower ion-diffusion resistance and accordingly exhibit outstanding high-rate capability and good cycle stability [14]. Therefore, constructing metal-selenide-based heterostructures would endow them with fascinating electrochemical performance. Fortunately, it has been found that cobalt selenites can transform into cobalt oxide and cobalt selenides in the initial charge/discharge processes and form metal oxide/selenide heterostructures. In these regards, cobalt selenites can be used as ideal and effective anodic materials for high-performance LIBs.

Nevertheless, cobalt selenites still suffer from pulverization due to the large volume change during the charge and discharge processes, leading to rapid capacity decay [16]. It is confirmed that the rational design of the structure of cobalt selenites is a practical strategy to overcome this problem [17,18,19]. For example, Jiang et al. prepared metastable CoSeO_3_‧H_2_O nanosheets, which could exhibit reversible capacities of 1100 and 515 mAh g^−1^ at 3 and 10 A g^−1^ after 1000 cycles, respectively [17]; anhydrous CoSeO_3_ porous microspheres were shown to be capable of delivering a high reversible capacity of 709 mAh g^−1^ after 1400 cycles at a current density of 3 A g^−1^ [16]. It is well-known that the morphologies of nanomaterials are dominated by the crystalline structure of initial seeds and external factors. As one kind of cobalt selenite, CoSeO_3_‧2H_2_O, has a different crystal structure from other cobalt selenites [17,20,21]. Thus, it is reasonable to consider that CoSeO_3_‧2H_2_O with a unique morphology would be obtained by rational design, and accordingly, the electrochemical performance would be improved. However, the synthesis and Li^+^ storage properties of CoSeO_3_‧2H_2_O have not been reported.

Recently, 3D hierarchical nanostructures assembled from low-dimensional building blocks have attracted tremendous attention in the field of LIBs [22]. As is known to all, 3D hierarchical nanostructures can not only provide large contact areas between the electrode and electrolyte, but also accommodate volume change and accelerate Li^+^ transport [23]. Hence, it is urgent to explore novel and effective methods for preparing hierarchical CoSeO_3_‧2H_2_O nanostructures to achieve the goals of high capacity and long life.

Herein, we report a simple and effective one-step hydrothermal method for the synthesis of hierarchical CoSeO_3_‧2H_2_O nanoflowers for the first time. The CoSeO_3_‧2H_2_O nanoflowers were assembled using nanosheets with thickness of ~10 nm. Owing to the unique structure, the obtained CoSeO_3_‧2H_2_O nanoflowers exhibited high specific capacity, superior rate capability, and excellent cycling stability.

## 2. Materials and Methods

### 2.1. Synthesis of Hierarchical CoSeO_3_‧2H_2_O Nanoflowers

All the chemicals were purchased from Shanghai Aladdin Bio-Chem Technology Co. Ltd. and used without purification. Co(CH_3_COOH)_2_‧4H_2_O (0.2491 g) and Na_2_SeO_3_‧5H_2_O (0.1315 g) were dissolved into a mixed solvent of 10 mL deionized water and 5 mL ammonium hydroxide (80%) and stirred for 10 min. Then, the obtained solution was transferred into an autoclave and heated at 150 °C for 12 h. After the reaction, the product was washed with deionized water and ethanol. After dried at 80 °C for 10 h, hierarchical CoSeO_3_‧2H_2_O nanoflowers were obtained. 

### 2.2. Structural Characterizations

The crystalline structure of the product was analyzed using an X-ray diffractometer (XRD, Rigaku D/max-2500, Rigaku Corporation, Tokyo, Japan) using Cu Kα radiation. The morphology, nanostructure, and composition of the sample were characterized using field emission scanning electron microscopy (FESEM, JEOL JSM-6700F, JEOL Ltd., Akishima, Tokyo, Japan), transmission electron microscopy (TEM, JEOL JEM-2010, JEOL Ltd., Japan), and high-resolution TEM (HRTEM, JEOL JEM-2010, JEOL Ltd., Japan).

### 2.3. Electrochemical Performance Measurement

Hierarchical CoSeO_3_‧2H_2_O nanoflowers (70 wt%), carbon black (20 wt%), and polyvinylidene fluoride (PVDF, 10 wt%) were mixed into N-methyl-2-pyrrolidone to form a slurry, which was then uniformly coated on copper foils and dried at 120 °C for 12 h in vacuum to generate working electrodes. The loading amount of the electrode was about 2 mg cm^−2^. 1 mol L^−1^ LiPF_6_ dissolved in ethylene carbonate (EC) and diethyl carbonate (DC) (1:1 by volume) was employed as the electrolyte. Li foil and polypropylene membrane were chosen as the counter electrode and separator, respectively. Then, the CR2025 button batteries were assembled in an argon-filled glovebox. The LAND CT2001A battery tester (Shenglan Electronic Technology Co., Ltd, Dongguan, China) was used to evaluate the electrochemical properties of the obtained anode.

## 3. Results

**Figure 1:**
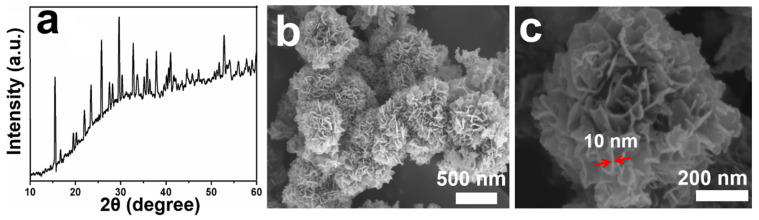
(**a**) XRD pattern, (**b**,**c**) FESEM images of the CoSeO_3_‧2H_2_O nanoflowers.

The XRD pattern of the sample is shown in Figure 1a. All diffraction peaks match well with monoclinic CoSeO_3_‧2H_2_O with space group of *P*2_1_/*n*(14) and lattice parameters of *a* = 6.5151 Å, *b* = 8.8253 Å, and *c* = 7.6404 Å (JCPSD No. 52-0215). The diffraction peaks at 15.48°, 25.71°, 29.60°, 32.73°, 35.81°, 37.83°, 40.95°, and 52.78° can be indexed to the (011), (012), (210), (031), (12−2), (113), (040), and (313) facets, respectively. Clearly, no other crystal phases (e.g., CoSe_2_, Co_3_O_4_) can be found, which reveals the high purity of the sample. 

The morphologies of the products were analyzed using FESEM, and the results are shown in Figure 1b,c. As can be seen from Figure 1b, the obtained products are consisted of nanoscale flower-like CoSeO_3_‧2H_2_O. As shown in the high-magnification FESEM image (Figure 1c), the CoSeO_3_‧2H_2_O nanoflowers are made up of numerous ultrathin nanosheets with thicknesses of ~10 nm (marked by the red arrows). In the synthesis, CoSeO_3_‧2H_2_O nuclei prefer to grow into ultrathin nanosheets owing to their anisotropic crystal characteristics. Thus, in the following growth process, to minimize the surface energies, these primary nanosheets have a strong tendency to interconnect with each other and form into 3D hierarchical nanoflowers with obviously open structure. This special hierarchical structure can accelerate the diffusion of Li^+^, provide more areas for the contact between electrode and electrolyte, and alleviate the volume changes during the charge/discharge processes, thus leading to enhanced electrochemical performance [24,25].

**Figure 2:**
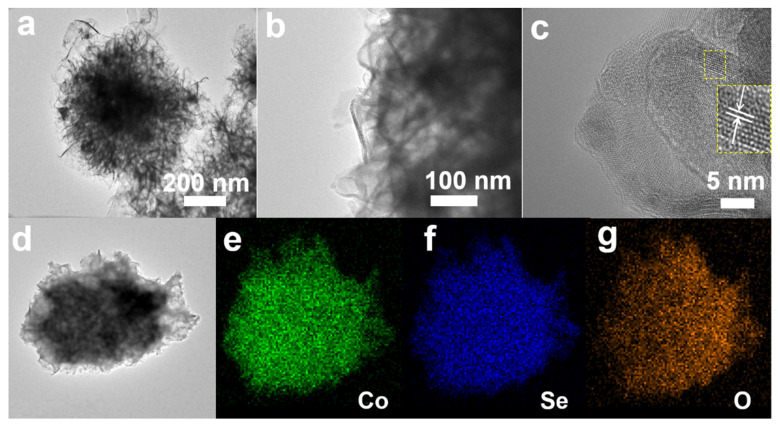
(**a**,**b**) TEM images, (**c**) HRTEM image, and (**d**) STEM image of the CoSeO_3_‧2H_2_O nanoflowers; (**e**–**g**) EDX mapping images of Co, Se, and O.

As shown in Figure 2, the nanostructures of the as-obtained CoSeO_3_‧2H_2_O nanoflowers were studied using TEM analysis. The different magnification TEM images (Figure 2a,b) can further prove that the samples are hierarchical nanoflowers assembled with ultrathin nanosheets. The HRTEM image (Figure 2c) shows the typical lattice spacing of 0.238 nm (marked by yellow dotted box), which agrees with the (113) facets of monoclinic CoSeO_3_‧2H_2_O. In addition, the element distribution of the CoSeO_3_‧2H_2_O nanoflowers were investigated using STEM (Figure 2d), coupled with EDX mapping (Figure 2e–g). Clearly, the Co, Se, and O elements are homogeneously distributed in the CoSeO_3_‧2H_2_O nanoflowers, coinciding with the above XRD result (Figure 1a).

**Figure 3:**
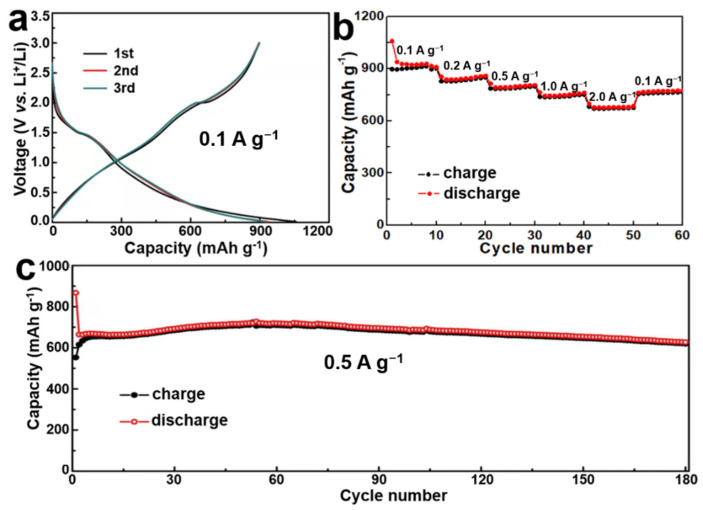
Electrochemical properties of the CoSeO_3_‧2H_2_O nanoflowers: (**a**) charge-discharge curves for the first three cycles at a current density of 0.1 A g^−1^; (**b**) rate capability at various current densities; (**c**) cycling performance at 0.5 A g^−1^.

The electrochemical properties of the obtained CoSeO_3_‧2H_2_O anode were researched through the galvanostatic method. Figure 3a shows the first, second, and third charge–discharge curves of the hierarchical CoSeO_3_‧2H_2_O nanoflower anodes in the voltage range of 0.01–3.0 V (vs. Li^+^/Li) at a current density of 0.1 A g^−1^. The initial discharge and charge capacities are 1064.1 and 897.3 mAh g^−1^, respectively, and the coulombic efficiency and the irreversible capacity of the first cycle is 84.3% and 166.8 mAh g^−1^. The capacity loss may be attributed to the formation of Li_2_O by intercalated Li^+^ and SEI film owing to the decomposition of electrolyte during the first cycle on the electrode surface [17]. In the following second and third cycles, the coulombic efficiency is 95.7% and 97%, respectively, which indicates the good reversibility of the CoSeO_3_‧2H_2_O anode.

The rate capability of the CoSeO_3_‧2H_2_O electrode was studied by progressively increasing the current densities from 0.1 to 0.2, 0.5, 1.0, and 2 A g^−^^1^. As shown in Figure 3b, the discharge capacity of the CoSeO_3_‧2H_2_O nanoflowers gradually decreases from 1058.9 to 858.1, 816.7, 765.5 and 678.6 mAh g^−^^1^ when increasing the current density from 0.1 to 0.2, 0.5, 1.0 and 2.0 A g^−^^1^, respectively. Importantly, the discharge capacity is still as high as 678.6 mAh g^−^^1^ as the current density is increased to 2.0 A g^−^^1^. Then, the discharge capacity can return to 762.7 mAh g^−^^1^ as the current density is reduced to 1 A g^−^^1^. The above results suggest the excellent structural stability at high current density and superior rate performance of the anode.

Besides the rate capability, the cycling performance of the CoSeO_3_‧2H_2_O electrode was also evaluated at a constant current density of 0.5 A g^−^^1^ for 180 cycles, and the result is shown in Figure 3c. As can be clearly seen, the curve is almost a straight line, and the electrode can retain a high reversible capacity of 626 mAh g^−^^1^ after 180 cycles. Meanwhile, the high coulombic efficiency of 98% can be obtained in the subsequent cycles after the first few cycles, which further indicates the excellent cyclic stability of the CoSeO_3_‧2H_2_O electrode. Figure S1 is the FESEM image of the CoSeO_3_‧2H_2_O electrode after the cyclic stability test. Obviously, the hierarchical structure of the sample can be maintained even after long and intensive battery operation, suggesting the excellent electrochemical stability. In addition, the discharge capacity of the electrode is slightly increased from the first to the fiftieth cycle and then slowly decreased during the following cycles, which may be attributed to the gradual electrolyte penetration and the reversible growth of the electrochemistry active polymeric gel-like film by the activated electrolyte degradation [26,27]. Compared to other Co, Se-based anodes materials [12,28,29,30,31,32,33,34,35], the CoSeO_3_‧2H_2_O nanoflowers exhibit better electrochemical performance (Table S1), suggesting their promising application in energy storage devices.

Based on some important literature [17,18,36], CoSeO_3_‧2H_2_O can be converted into CoO and SeO_2_ during the first discharge and charge processes. In the following lithiation and delithiation procedures, the reversible reaction mechanism of CoO and SeO_2_ with Li^+^ ions can be described by the reaction (Equation (1)):

CoO + *x*SeO_2_ + (1 − *x*)Se + 4(*x* + 1)Li^+^ + 4(*x* + 1)e^−^ ↔ Co + (2*x* + 1)Li_2_O + Li_2_Se. (1)

In order to better comprehend the Li^+^-ions storage mechanism of CoSeO_3_‧2H_2_O, some analysis methods, such as in situ EIS, in situ XRD, ex suit XPS, ex suit TEM, and so on, can be further conducted [36,37]. On the other hand, to deeply research the relationship between nanostructure and performance, it is appropriate to fabricate other control samples in the future. In addition, it is worth mentioning that the improvement of low-temperature performance is very important for next-generation LIBs [38]; furthermore, size reduction, doping, and surface modification are potential methods for enhancing the low-temperature performance of electrodes [39,40]. Hence, it is reasonable to consider that many efforts should be made to the controlled synthesis of CoSeO_3_‧2H_2_O with other morphologies, heteroatoms doped CoSeO_3_‧2H_2_O, and CoSeO_3_‧2H_2_O@C composites.

## 4. Conclusions

In summary, CoSeO_3_‧2H_2_O nanoflowers were successfully synthesized using an effective and simple hydrothermal method for the first time. When used as the anode materials for LIBs, the CoSeO_3_‧2H_2_O electrodes exhibited excellent Li^+^ storage properties, which can be attributed to the hierarchical flower-like architecture and the open structure. The present method is expected to be extended to the synthesis of other metal selenites with unique morphologies and improved properties.

## Data Availability

Data presented in this article are available at request from the corresponding author.

## Supplementary Materials

The following supporting information can be downloaded at: https://www.mdpi.com/article/10.3390/nano12142474/s1, Figure S1: FESEM image of the CoSeO_3_‧2H_2_O electrode after 180 cycles at 0.5 A g^−1^; Table S1: The comparison of the electrochemical performance the CoSeO_3_‧2H_2_O nanoflowers and other previously reported Co,Se-based anodes for LIBs. References [12,28,29,30,31,32,33,34,35] are cited in the Supplementary Materials.
