# Supplementary material for: Facile Synthesis of Hierarchical CoSeO3‧2H2O Nanoflowers Assembled by Nanosheets as a Novel Anode Material for High-Performance Lithium-Ion Batteries
Source: Nanomaterials (Basel). 2022 Jul 19;12(14):2474. doi: 10.3390/nano12142474 (PMC9320587; doi:10.3390/nano12142474)
Supplement: Supplementary file 1 [file nanomaterials-12-02474-s001.zip › nanomaterials-1784963-supplementary.pdf]

nanomaterials

MDPI

Communication

Facile Synthesis of Hierarchical $CoSeO_3·2H_2O$ Nanoflowers Assembled by Nanosheets as a Novel Anode Material for High-Performance Lithium-ion Batteries

Xiao-Xu Ji [1], Qing-Huai Zhao [1], Hao Chen [2], Xin-Wei Luo [2], Yi Shang [2] and Xiao-Di Liu [2,*]

1 College of Physics and Electronic Engineering, Nanyang Normal University, Nanyang, 473061, China; xxji2010@163.com (X.-X.J.); zqh1262022@126.com (Q.-H.Z.);
2 College of Chemistry and Pharmaceutical Engineering, Nanyang Normal University, Nanyang, 473061, China; xiaohao819@126.com (H.C.); 17527755065@163.com (X.-W.L.); s3030199101@163.com (Y.S.);
* Correspondence: 20122029@nynu.edu.cn

Abstract: As novel anodic materials for lithium-ion batteries (LIBs), transitional metal selenites can transform into metal oxide/selenide heterostructures in the first cycle, which can help to enhance the Li^+ storage performance, especially high discharge capacity. Herein, well-defined hierarchical $CoSeO_3·2H_2O$ nanoflowers assembled by 10-nm-thick nanosheets are successfully synthesized *via* a facile one-step hydrothermal method. When used as anodic material for LIBs, the $CoSeO_3·2H_2O$ nanoflowers exhibit a considerable high discharge capacity of 1064.1 mAh g^{-1} at a current density of 0.1 A g^{-1}. In addition, the obtained anode possesses good rate capability and cycling stability. Owing to the superior electrochemical properties, the $CoSeO_3·2H_2O$ nanoflowers would be served as promising anodic materials for high-performance LIBs.

Keywords: hydrothermal method; $CoSeO_3·2H_2O$; nanoflowers; nanosheets; lithium-ion batteries

Citation: Ji, X.-X.; Zhao, Q.-H.; Chen, H.; Luo, X.-W.; Shang, Y.; Liu, X.-D. Facile Synthesis of Hierarchical $CoSeO_3·2H_2O$ Nanoflowers Assembled by Nanosheets as a Novel Anode Material for High-Performance Lithium-ion Batteries. *Nanomaterials* **2022**, *12*, 2474. https://doi.org/10.3390/10.3390/nano12142474

Academic Editor: Cheol-Min Park

Received: 8 June 2022
Accepted: 18 July 2022
Published: 19 July 2022

Publisher's Note: MDPI stays neutral with regard to jurisdictional claims in published maps and institutional affiliations.

Copyright: © 2022 by the authors. Submitted for possible open access publication under the terms and conditions of the Creative Commons Attribution (CC BY) license (https://creativecommons.org/licenses/by/4.0/).

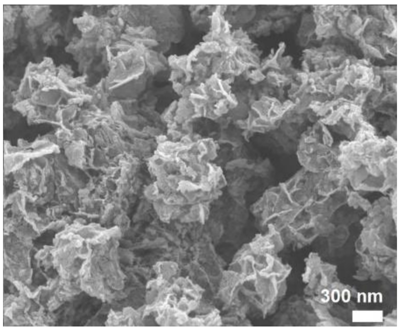

Figure S1. FESEM image of the $CoSeO_3{\cdot}2H_2O$ electrode after 180 cycles at 0.5 A g^{-1}.

Table S1. The comparison of the electrochemical performance the $CoSeO_3{\cdot}2H_2O$ nanoflowers and other previously reported Co,Se-based anodes for LIBs.

Sample	Discharge Capacity	Cyclability	Ref.
$CoSeO_3{\cdot}2H_2O$ nanoflowers	765.5 mAh g^{-1}(1 A g^{-1})	626 mAh g^{-1}/180th (0.5 C)	this work
$CoSe_2$ decorated $NbSe_2$ nanosheets	466 mAh g^{-1}(1 A g^{-1})	364.7 mAh g^{-1}/1500th (5 C)	[1]
$CoSe_2$@MXene	763 mAh g^{-1}(1 A g^{-1})	910 mAh g^{-1} /100th (0.2 C)	[2]
ZnSe/$CoSe_2$@N-C composites	399.6 mAh g^{-1}(1 A g^{-1})	755mAh g^{-1}/100th (0.1 C)	[3]
ZnSe/$CoSe_2$-C	579 mAh g^{-1}(0.8 A g^{-1})	700 mAh g^{-1}/500th (1 C)	[4]
Mesoporous hollow Co_3O_4 hierarchical architecture	629.4 mAh g^{-1}(1 A g^{-1})	555.7 mAh g^{-1}/1000th (1 C)	[5]
Porous Co_3O_4 spheres	399.6 mAh g^{-1}(1 A g^{-1})	230 mAh g^{-1}/1000th (2 C)	[6]
Urchin-like $CoSe_2$	390 mAh g^{-1}(1 A g^{-1})	410 mAh g^{-1}/1800th (1 C)	[7]
$NiCo_2O_4$ Hollow Spheres	662 mAh g^{-1}(1 A g^{-1})	706 mAh g^{-1}/100th (0.2 C)	[8]
$MoSe_2$/MoO_2 Composite	741 mAh g^{-1}(1 A g^{-1})	547 mAh g^{-1}/300th (2 C)	[9]

References:

[1] Zhang, J.L.; Du, C.F.; Zhao, J.; Ren, H.; Liang, Q.H.; Zheng Y.; Madhavi, S.; Wang, X.; Zhu, J. W.; Yan, Q. Y. $CoSe_2$-decorated $NbSe_2$ nanosheets fabricated via cation exchange for Li storage. *ACS Appl. Mater. Inter*. **2018**, *10*, 37773–37778.

[2] Hong, L.; Ju S.L.; Yang, Y.H.; Zheng, J.N.; Xia, G.L.; Huang, Z.G.; Liu, X.Y.; Yu, X. B. Hollow-shell structured porous $CoSe_2$ microspheres encapsulated by MXene nanosheets for advanced lithium storage. *Sustain. Energy Fuels* **2020**, *4*, 2352–2362.

[3] Hu, X.J.; Liu, X.J.; Chen, K.; Wang, G.; Wang, H. Core-shell MOF-derived N-doped yolk-shell carbon nanocages homogenously filled with ZnSe and $CoSe_2$ nanodots as excellent anode materials for lithium- and sodium-ion batteries. *J. Mater. Chem. A* **2019**, *7*, 11016–11037.

[4] Wang, K.; Wang, Y.P.; Zhang, Y.F.; Liu, F.; Shi, J.R.; Liu, S.Y.; Xie, X.F.; Cao, G.Z.; Pan, A.Q. Bimetallic organic framework derivation of three dimensional and heterogeneous metal selenides/carbon composites as advanced anodes for lithium-ion batteries. *Nanoscale* **2020**, *12*, 12623–12631.

[5] Wang, G.Y.; Zhang, M.; Deng, Z.P.; Zhang, X.F; Huo, L.H.; Gao, S. Poplar branch bio-template synthesis of mesoporous hollow Co_3O_4 hierarchical architecture as an anode for long-life lithium ion batteries. *Ceram. Int*. **2020**, *46*, 29033–29040.

[6] Feng, Y.; Cao, C.Y.; Zeng, J.; Wang, R.C.; Peng, C.Q.; Wang, X.F. Hierarchical porous Co_3O_4 spheres fabricated by modified solvothermal method as anode material in Li-ion batteries. *Trans. Nonferrous Met. Soc. China* **2022**, *32*, 1253–1260.

[7] Zhang, K.; Park, M.; Zhou, L.; Lee, G.H.; Li, W.; Kang, Y.M; Chen, J. Urchin-like $CoSe_2$ as a high-performance anode material for sodium-ion batteries. *Adv. Funct. Mater*. **2016**, *26*, 6728–6735.

[8] Shen, L.F.; Yu, L.; Yu, X.Y.; Zhang, X.J.; Lou, X.W. Self-templated formation of uniform $NiCo_2O_4$ hollow spheres with complex interior structures for lithium-ion batteries and supercapacitors. *Angew. Chem. Int. Ed*. **2015**, *54*, 1868–1872.
[9] Hao, Q.Y.; Cui, G.L.; Zhao, Y.; Bakenov, Z. Flower-like $MoSe_2/MoO_2$ composite with high capacity and long-term stability for lithium-ion battery. *Nanomaterials* **2019**, *9*, 1256.
